# Exercise-induced increase in brain-derived neurotrophic factor in human Parkinson's disease: a systematic review and meta-analysis

**DOI:** 10.1186/s40035-018-0112-1

**Published:** 2018-03-20

**Authors:** Mark A. Hirsch, Erwin E. H. van Wegen, Mark A. Newman, Patricia C. Heyn

**Affiliations:** 1 Carolinas Medical Center, Carolinas Rehabilitation, Department of Physical Medicine and Rehabilitation, 1100 Blythe Blvd, Charlotte, NC 28203 USA; 2 Department of Rehabilitation Medicine, Amsterdam Movement Sciences/Amsterdam Neurosciences, VU University Medical Center, PO Box 7057, 1007 Amsterdam, MB The Netherlands; 3 Department of Physical Medicine and Rehabilitation, Anschutz Medical Campus, University of Colorado, Denver, USA

**Keywords:** Brain derived neurotrophic factor, Exercise, Rehabilitation, Systematic review, Parkinson’s disease

## Abstract

**Background:**

Animal models of exercise and Parkinson’s disease (PD) have found that the physiologic use of exercise may interact with the neurodegenerative disease process, likely mediated by brain derived neurotrophic factor (BDNF). No reviews so far have assessed the methodologic quality of available intervention studies or have bundled the effect sizes of individual studies on exercise-induced effects on BDNF blood levels in human PD.

**Research design and methods:**

We searched MEDLINE, EMBASE, Cochrane Library, PsycINFO and PubMed from inception to June 2017.

**Results:**

Data aggregated from two randomized controlled trials and four pre-experimental studies with a total of 100 ambulatory patients with idiopathic PD (Hoehn/Yahr ≤3) found improvements in BDNF blood concentration levels in all 6 studies (two RCTs and 4 pre-experimental studies). Pooled BDNF level change scores from the 2 RCTs resulted in a significant homogeneous summary effect size (Standardized Mean Difference 2.06, 95% CI 1.36 to 2.76), and a significant heterogeneous SES for the motor part of the UPDRS-III examination (MD -5.53, 95% CI -10.42 to -0.64). Clinical improvements were noted in all studies using a variety of outcome measures.

**Limitations:**

The evidence-base consists primarily of small studies with low to moderate methodological quality.

**Conclusions:**

This review provides preliminary evidence for the effectiveness of physical exercise treatments for persons with PD on BDNF blood levels. Further research is needed.

**Electronic supplementary material:**

The online version of this article (10.1186/s40035-018-0112-1) contains supplementary material, which is available to authorized users.

## Background

Parkinson’s disease (PD) is a complex, chronic, disabling neurodegenerative condition for which there is no cure [1]. The incidence of PD is expected to double in the next 15 years. The motor features of the disease include bradykinesia, rigidity, tremor, gait impairment and postural instability. Non-motor features include cognitive impairment, depression, sleep problems, osteoporosis, anxiety, fatigue and constipation. Increasingly, evidence supports efficacy of physical therapy and physical exercise interventions as adjunctive (i.e., helpful) to dopamine replacement therapy for control of motor symptoms and non-motor features, with improved quality of life for people at all stages of PD [2–9]. The physiologic effects of exercise may impact a number of plasticity-related events in PD brain including synaptogenesis, angiogenesis, and neurogenesis [10, 11].

In rodent PD models, physical exercise was found to interact with the neurodegenerative process [12–14], likely mediated by use-dependent expression of endogenous neurotrophic factors [5, 15–28]. The scientific evaluation of exercise induced changes in brain-derived neurotrophic factor (BDNF) concentration is emerging as a key research area in healthy adult populations [29–31] and in neurodegenerative populations (e.g., multiple sclerosis [32, 33]) (schizophrenia [34, 35]). Endogenous production of BDNF by voluntary exercise was shown in adult rats [36], and is purported to play a crucial role in neuroplastic effects of rehabilitation interventions of humans with neurodegenerative disease [28, 29, 37–40].

The physiologic mechanisms underlying exercise-induced BDNF changes are not well understood in PD but could include long-term potentiation and long-term depression mechanisms [41–43]. BDNF and exercise both promote survival and growth of neurons in pars compacta of substantia nigra and recovery of motor behavior [44]. In the 6-hydroxydopamine model of PD and exercise, blocking of BDNF receptors causes enhanced postlesion nigrostriatal dopaminergic cell loss, quantified as a reduction in the expression of tyrosine hydroxylase (TH), a rate-limiting enzyme in dopamine biosynthesis [22, 45]. Additionally, BDNF may ameliorate neuronal dysfunction and neurodegeneration by modulating 1-methyl-4-phenylpyridinium (MPP+)-induced neurotoxicity [46], pathologic brain mitochondria function [47], or DNA repair by stimulating transcription factors such as CREB (cyclic AMP response element-binding protein) [48].

Recent reports by the Movement Disorder Society (MDS) Evidence-Based Medicine Panel on non-pharmacologic interventions for PD and the European Physiotherapy Guideline Development Group Panel recommended that future studies ought to focus on exercise-induced neuroplasticity in humans with PD [49, 50]. To the best of our knowledge, no reviews so far have assessed the methodologic quality of available intervention studies or have bundled the effect sizes of individual studies on exercise-induced changes in BDNF blood levels in human PD. The objective of this review was to systematically identify and appraise the evidence, methodological quality and clinical outcomes of intervention studies on the effects of physical exercise on endogenous production of BDNF in human PD, to bring such insights into the clinical context of rehabilitation for people living with PD.

## Method

### Data sources and search strategy

This study was conducted in accordance with the Preferred Reporting Items for Systematic Reviews and Meta-Analyses (PRISMA) statement [51]. An a priori protocol [52] was adhered to throughout the review process to minimize risk of bias. An electronic literature search was conducted independently by one of the authors (PH) and a research assistant in the following databases: Medline (Ovid), PubMed (NLM), Embase (Embase.com), PsycINFO (Ovid), Physiotherapy Evidence Database (PeDro) and the Cochrane library (Wiley). We included the following key words (including MESH): Parkinson’s, Parkinson’s disease AND exercise, exercise training, physical activity, therapy, physical therapy, physical exercise, physical training, exercise-induced, exercise-enhanced AND human, people, person, individual, patient, older, elderly, AND neurotrophic factor, growth factor, brain derived neurotropic factor, neuroplasticity, plasticity, AND trial, intervention, training, treatment OR control, controlled, randomized.

### Criteria for inclusion

We exclusively focused on studies evaluating the effects of exercise interventions on brain-derived neurotrophic factor in patients with PD. Studies were accepted when: 1) they used human participants with diagnosis of PD, 2) they used a prospective intervention design with or without a control group, 3) they contained physical exercise training or a physical exercise intervention component, 4) they assessed neurotrophic factor(s), 5) they were written in English, 6) they were published in a peer-reviewed journal. Non-human studies, non-physical exercise trials, grey literature, studies using mixed populations, single case studies, studies not specific to PD, and studies without assessment of neurotrophic factors were excluded. The search was conducted up to June 2017.

### Review levels and data extraction

**Fig. 1:**
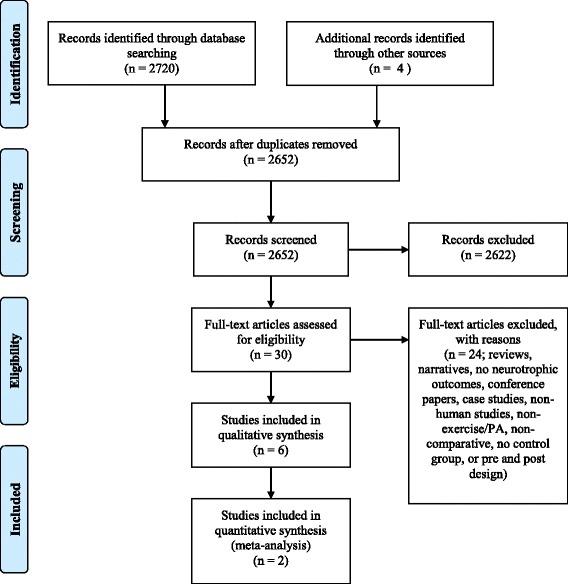
PRISMA flow diagram

Initial citation screening (MH, PH) was based on reviewing title and abstract (Level 1 Review) of all database search hits. A second round was implemented (Level 2 review) in which three independent reviewers (EvW, MN, MH) analysed the full manuscripts and performed additional reference tracking. A total of 30 papers were imported into a widely used, web-based, production platform system for reviews (www.covidence.org). Disagreements were resolved in a consensus meeting (Fig. 1). Six manuscripts passed onto full data extraction (LEVEL 3 review in covidence.org). Data on study design, sample size and characteristics, exercise dosing, clinical outcome measures and laboratory results) were extracted manually by two independent data abstractors (MH, MN) and summarized in Table 1. Authors of relevant publications were contacted for data when post intervention means and/or SDs were not reported. When two or more randomized clinical trials were available reporting on the same outcomes, quantitative meta-analysis (i.e., pooling using Hedges’ g) of the findings was performed using Cochrane methodology, in Review Manager 5.3 [53].

**Table 1:** Exercise-induced BDNF response in human PD and clinical outcome

Author Country Year [PMID]	N Age PD characteristics setting	Outcome measures	Protocol	Results	
				Pre to post exercise BDNF level mean±SD and BDNF Effect Size	Clinical measures score and UPDRS Effect Size
Sajatovic USA 2017 [28579759]	28 Exp=69.8±9.3 yrs Con=70.3±6.5 H&Y ≤3 6.8±5.3 yrs since diagnosis OR	pBDNF MADRS MoCA UPDRS III SCOPA	Interval high-cadence cycling 3 sessions per week for 45-60 minutes each session for 12 weeks. High cadence interval stationary cycling (20 min) at 60-80% Hear Rate maximum. Resistance training 2-4 sets of 8-12 repetitions for each set. Self-management exercise 3 times per week (SGE) and psychoeducation (12 60 minute group sessions).	T0 (baseline) 26.8±15.6 pg/mL T1 (12 weeks, post test) 90±166.4 pg/mL T2 (24 weeks) 38.5±46.2 pg/mL 335.8% ↑ pBDNF level at T1 (12 weeks, post-test, p<0.001)	MADRS at T0 21.2±6.3 MADRS at T1 15.2±8.0 Δ28.3% ↓ MADRS MADRS at T2 14.2±8.5 Δ33.0% ↓ MADRS MoCA at T0 23.3±4.1 MoCA at T1 25.2±3.7 Δ7.5% ↑ MoCA MoCA at T2 25.2±5.1 Δ0% ↑ MoCA SCOPA-Sleep (night sleep) at T2 12.1±4.1 Δ14.0% ↑ SCOPA-Sleep (night sleep) MADRS, MoCA, an SCOPA-Sleep (night sleep) (all p<0.01).
Frazzitta Italy 2014 [24213955]	24 Exp=67±5 yrs pf age Con=65.4±4 yrs of age H&Y 1-1.5 8±3.5 yrs since diagnosis IR	sBDNF UPDRS III BBS 6MWT	Physical therapy 15 X 60 minute sessions per week/ 3 sessions per day Exercise on treadmill, 30 minutes per session, 5 days/week at ≤60% HRR and a maximum speed of 3.5 km/h for 4 weeks	T0 (admission) 21.64±3.4 ng/mL T1 (10 days) 25.04±7.3 ng/mL T2 (20 days) 25.79±7.9 ng/mL T3 (discharge) 24.77±6.4 ng/mL ES of ΔsBDNF=1.1 (p<0.0001) 12.6% ↑ sBDNF level at T3.	UPDRS III at T0 16.4±3.5 UPDRS III at T3 8.8±3.2 ES of ΔUPDRS III = -3.3 Δ46.3% ↓UPDRS III UPDRS II at T0 8.14±3.3 UPDRS II at T3 5.50±3.0 Δ32.4% ↓UPDRS II BBS at T0 48.64±6.1 BBS at T3 54.00±2.4 Δ9.9% ↑BBS 6MWT (m) at T0 383±94 6MWT at T3 477±79 Δ19.7% ↑6MWT distance ΔUPDRS II, BBS, and 6MWT (all p<0.01). No statistical association between BDNF levels and clinical measures.
Marusiak Poland 2015 [25510618]	11 71±10 yrs of age H & Y 1-3 8±4 yrs since diagnosis OR	sBDNF UPDRS III Myometry	Exercise using stationary bicycle, 3 x per wk, 60 minutes per session for 8 weeks	34% ↑ sBDNF level at post-test (p<0.05). No sBDNF level change in healthy controls (p=0.809). **Within Group Effect Size BDNF Serum Level: PD: BDNF T0-T1: 0,95 (CI -2,38-1,58)** **CONTROL: BDNF T0-T1: 0,10 (CI -1,22-2,33)**	↑ sBDNF level correlated with improvements in PD rigidity (p<0.05). **Between Groups Effect Size: Not applicable because controls are healthy.**
Angelucci Italy 2016 [26863448]	9 62.7±6.8 yrs of age 11.78±7.3 yrs since diagnosis IR	sBDNF UPDRS II UPDRS III 6MWT PDQ-39	Physical therapy 3 session/day/5 days per week for 30 days Exercise on treadmill, 20 minutes per session, 5 days per week at 3.5-4 km/h at ≤60% HRR Exercise using stationary bicycle at 25-30 km/h Exercise using Wii system Fit Balance board	T0 (admission) 2171.03±1699.69 pg/mL T7 (7 days) 3396.78±1359.56 pg/mL T14 (14 days) 2670.01±1439.64 pg/mL T21 (21 days) 2387.38±1088.84 pg/mL T30 (30 days) 2339.31±1666.01 pg/mL 36.09% ↑ sBDNF level T7 (p<0.01). No statistically significant changes in sBDNF at 14d, 21d, 30d, 90d **Within Group Effect Size BDNF Serum Level: BDNF T0-T7 = 0,84 (CI 0,04-1,96)** **BDNF T0-T14 = 0,34 (CI 0,60-1,45)** **BDNF T0-T21 = 0,16 (CI 0,55-1,27)** **BDNF T0-T30 = 0,11 (CI 0,98-1,28)** **BDNF T90 days data is missing.**	UPDRS II T0 21.77±3.38 UPDRS II T30 7.55±3.12 Δ65.3% ↓UPDRS II UPDRS III T0 31.62±10.68 UPDRS III T30 14.44±8.07 Δ24.4% ↓UPDRS III UPDRS III G&B score T0 6.74±5.21 UPDRS III G&B score T30 2.33±1.32 Δ65.4% ↓UPDRS III G&B score 6MWT (m) T0 252.33±111.81 6MWT T30 374.22±124.62 Δ32.5% ↑6MWT distance PDQ-39 T0 61.0±23.5 PDQ-39 T30 45.44±21.01 Δ25.5% ↓PDQ-39 (all p<0.01 at T30). **Within Group Effect Size UPDRS-IIl: UPDRS-III T0-T30 = -1,93 (CI -7,20 to -5,05)**
Fontanesi Italy & USA 2016 [26253177]	16 71.5±6.8 yrs of age 8.43±4.08 yrs since diagnosis IR	BDNF-TrkB UPDRS 6MWT BBS TUG PDDS FOGQ	physical and occupational therapy, 3 session a day, 5 days per week, for 4 weeks Multimodal exercise (i.e., treadmill with visual and auditory cueing, stationary bicycle, eliptical machine, stabilometric platform).	T1 (30 days) 52.6% ±10.8% ↑ in sBDNF-tyrosine receptor kinase signaling (p<0.001). **No data on BDNF concentration.**	UPDRS total score at T0 (baseline) 43.31±11.99 UPDRS total score at T1 29.56±9.46 Δ31.7% ↓UPDRS total score UPDRS III at T0 19.31±4.71 UPDRS III at T1 13.06±3.28 Δ32.3% ↓UPDRS III 6MWT at T0 292.38±103.46 6MWT at T1 363.63±114.64 Δ20% ↑in 6MWT distance BBS at T0 44.74±7.21 BBS at T1 52.50±4.18 Δ14.7% ↑BBS score PDDS at T0 71.13±14.53 PDDS at T1 54.25±12.12 Δ23.7% ↓PDDS score FOGQ at T0 14.27±5.22 FOGQ at T1 9.73±4.03 Δ31.8% ↓FOGQ score (all p<0.01) **Within Group Effect Size UPDRS-IIl: UPDRS-III T0-T30 =** **-0,63 (CI -5,72 to -2,29)**
Zoladz Poland 2014 [24930517]	12 70 ± 3 yrs of age H&Y 1-3 8.5± 1.3 yrs since diagnosis OR	sBDNF UPDRS	Interval exercise on a stationary bicycle, 3 x 60 minute sessions per week, for 8 weeks. 8 sets of 5 minute intervals including 3-minutes at 80-90 rpm and 2-minutes at less than 60 rpm. Target heart rate to achieve during cycling exercise was calculated as 60-75% of the Maximum Heart Rate.	T0 (baseline) 10977±756 pg/mL T1 (60 days) 14206±1256 pg/mL Δ 22.7% ↑ in sBDNF levels in PD (p<0.05). **Within Group Effect Size BDNF Serum Level: BDNF T0-T1 = 3,25 (CI 2,54-3,68)**	UPDRS total score at T0 48.9±4.3 UPDRS total score at T1 38.1±3.9 Δ 22% ↓ UPDRS total score (p<0.05). **Within Groups Effect Size UPDRS-III: no data.**

Table Note: *sBDNF* serum brain derived neurotrophic factor, *pBDNF* plasma brain derived neurotrophic factor, *UPDRS* Unified Parkinson’s disease Rating Scale, UPDRS part III (motor examination), UPDRS part II (Activities of daily living), *UPDRS G&B* UPDRS gait and balance score, *MADRS* Montgomery–Asberg Depression Rating Scale, *MoCA* Montreal Cognitive Assessment, *SCOPA* Scales for Outcomes in PD – Sleep, *BBS* Berg Balance Scale, *6MWT* six minute walk test, *PDQ-39* Parkinson’s disease Quality of Life test 39 questions, *d* days, *wks* weeks, *yrs* years, *Exp* experimental group, *Con* control group, *H & Y* Hoehn and Yahr stage of Parkinson’s disease, *IR* inpatient rehabilitation, *OR* outpatient rehabilitation, *N* sample size, *PMID* pub med identification number, *HRR* hear rate reserve, *rpm* revolutions per minute, *km/h* kilometers per hour, *d* days; *Moyometry* myometric quantification using MyotonPRO myometry (Myoton AS, Tallinn, Estonia), *TUG* timed-up-and-go test, *PDDS* Parkinson’s disease disability scale, *FOGQ* Freezing of Gait Questionnaire, *TrkB* tyrosine receptor kinase, *sVCAM-1* Basal serum soluble vascular cell adhesion molecule-1, *TNF-α* basal serum tumor necrosis factor, *ES* effect size, *SD* standard deviation, *con* control group, *exp* experimental group

‘Exercise’ defined as “a subcategory of physical activity that is planned, structured, repetitive, and purposive in the sense that the improvement or maintenance of one or more components of physical fitness is the objective” [87] (pg. 250). The effect sizes for studies without a control group should be interpreted with caution for the following reasons: a) we do not control for dependency between outcomes in an uncontrolled pre-post design with our classical Cohen’s d, b) we have no control group data on the correlation between pre and post measurements, and c) from studies using a pre-experimental design we cannot rule out bias/placebo effects [88]

### Critical appraisal method

Appraisal of individual study methodological quality was based on published quality assessment tools developed jointly by methodologists from NHLBI and Research Triangle Institute International (www.nhlbi.nih.gov/health-pro/guidelines/in-develop/cardiovascular-risk-reduction/tools).

The NHLBI Appraisal tools (Additional file 1) includes items for evaluating the internal validity, descriptive criteria and statistical criteria of studies (Additional file 1). A quality rating (‘good’, ‘fair’, ‘poor’) was adapted from the review by Lim et al. [54]. Studies were considered to be of ‘good’ quality if at least 80% of the criteria were met; ‘fair’ quality when 51% to 79% of the criteria were met, and ‘poor’ quality when less than or equal to 50% of the criteria were met. Separate lists of methodological quality criteria were used for randomized controlled trials, uncontrolled pre-post studies and case control studies (Additional file 1).

Two evaluators (MH, MN) independently rated the methodological quality of the included studies using the NHLBI appraisal tools. Next, a kappa statistic was calculated for descriptive purposes and to investigate the agreement between the two evaluators on each appraisal tool (Additional file 1). The kappa values were interpreted using the criteria suggested by Tooth and Ottenbacher [55], <.40 poor agreement, .40 to .60 fair or moderate agreement, .60 to .80 good agreement, and >.80 perfect or excellent agreement. Additionally, we report the exact agreement among the two evaluators before disagreements about scoring were discussed (Additional file 1). Disagreements about scoring were resolved through discussion. If no consensus was reached, a third reviewer (EvW) made the final decision.

### Effect size analysis

For individual RCT’s, we calculated the difference between the pre-to the post-intervention change scores for experimental and control groups. In case of MD-UPDRS-III, we used the mean difference (MD) between the change scores because the same outcome measure was assessed in the trials. For BDNF, reported as serum [56] and plasma levels [57], we used the standardized mean difference (SMD) based on Hedges’ g by calculating the MD, divided by the average population standard deviation (SDi). The MD or SMD values of individual studies were averaged (pooled), resulting in a summary effect size (SES) with corresponding 95% confidence interval (CI). Following Cohen [58] we classified effect sizes into small (<0.2), medium (0.2-0.8), and large (>0.8). The I^2^ statistic was calculated to determine between-study variation [59]. In case of statistical heterogeneity (I^2^ ≥50%,) we applied a random-effects model. For I^2^ <50 % a fixed-effect model was applied.

Two studies, one prospective study on exercise-induced changes in BDNF tyrosine receptor kinase signaling (BDNF-TrkB) [60] and the prospective study by Zoladz and colleagues [61], were excluded from the BDNF level effect size analysis. The study by Fontanesi et al. [60] was excluded from the BDNF meta-analysis analysis because the authors did not report serum or plasma BDNF levels but instead reported BDNF tyrosine receptor kinase signaling. The study by Zoladz et al. [61] was excluded from the BDNF meta-analysis analysis because there was significant overlap in the study participant groups included in the paper by Zoladz et al. [61] and the study participants included in the paper by Marusiak et al. [62] (personal communication with Dr. Marusiak). The more recent candidate paper by Marusiak et al. [62] with the larger sample size (11 healthy controls and 11 patients with PD) was chosen for inclusion.

## Results

### Summary of the literature

The subject demographic characteristics, study design, exercise dosing, outcome measures and results are described in Table 1. A total of 100 participants contributed to the studies reported in this review. For the evaluation of physical exercise on BDNF levels, data were aggregated from two RCTs [56, 57] with a total of 52 ambulatory in- and outpatients with mild to moderate idiopathic PD severity, mean 7.0±1.5 years after PD diagnosis and 68±5.6 years (mean±standard deviation) of age at the time of study enrollment. BDNF concentration was assessed by enzyme-linked immunosorbent assay (ELISA) using standardized procedures at the completion of the 28-day intervention in the study by Frazzitta et al. [56] and at the completion of the 90-day intervention in the study by Sajatovic et al. [57]. Percent change BDNF levels were reported from two pre-experimental studies (Marusiak et al. [62], Angelucci et al. [63] Table 1), with a total of 20 ambulatory in- and outpatients with mild to moderate idiopathic PD severity (Hoehn and Yahr stage ≤3, range 1-3), 9.8±6.0 years (range 2-26 years) after PD diagnosis and 66.8±8.3 years of age at the time of study enrollment. BDNF concentration was assessed by ELISA using standardized procedures at the completion of the 30-day intervention in the study by Angelucci et al. [63] and at the completion of the 60-day intervention in the study by Marusiak et al. [62].

Clinical outcomes data were aggregated from two RCTs [56, 57]; and four pre-experimental studies [60–63] with a total of 100 ambulatory in- and outpatients with mild to moderate idiopathic PD severity (Hoehn and Yahr stage ≤3), 8.4±4.9 years after PD diagnosis, 68.7±6.8 years of age at the time of study enrollment. Methodological quality was scored for the six included studies [56, 57, 60–63] (Additional file 1).

Across all studies the participant characteristics were relatively homogenous in terms of years of age, PD stage, years since diagnosis, and ambulatory status. The studies were clinically homogenous with regards to including stationary cycling [57, 60–63] and administration of the MDS-UPDRS motor examination (Part III). Details about “on” or “off” state testing or training were not provided in the study by Fontanesi et al. [60] and Frazzitta et al. [56]. Physical exercise training, clinical outcome evaluations and BDNF testing procedures were conducted during the “on” phase in two studies [57, 63]. Zoladz et al. [61] and Marusiak et al. [62] administered outcome measures during the “off” phase. Differences among studies were noted regarding the use of elliptical machines [60], resistance training [57], physical therapy [56, 63], occupational therapy [60], treadmill training [56, 60, 63], Wii System Fit [63], and stabilometric platform [60]. All except one study reported exercise interventions being delivered by physiotherapists. Sajatovich et al. [57] used a Parkinson peer exercise leader to lead the exercise intervention. The setting for the interventions varied between laboratory [61, 62], hospital-based in-and out-patient [56, 60, 63] and fitness clinic-based [57]. The trial by Sajatovic et al. [57] was conducted in a community-based setting using a group versus an individual self-management program. In the study by Angelucci et al. [63] participants exercised in a group setting. None of the other studies reported details whether a group exercise intervention or an individual approach exercise intervention was used.

### BDNF levels

**Fig. 2:**
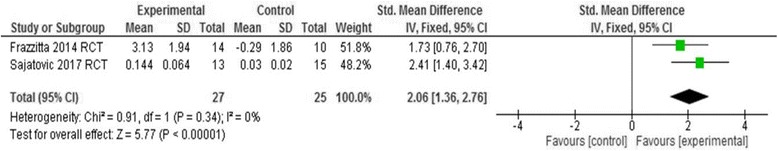
Summary effect sizes for outcome of change in BDNF levels. Green squares indicate individual SES. Black colored diamond indicates the summary effect size; *RCT* randomized clinical trial, *SD* standard deviation, *Std* standardized, *CI* Confidence Interval, *I*^2^ statistic to determine heterogeneity, *Z* z-score

BDNF levels assessed with laboratory measures were reported in 2 RCTs [56, 57] (N = 52) and pooling resulted in a significant homogeneous SES (SMD 2.06, 95% CI 1.36 to 2.76; Z = 5.77, *P* < .000001, I^2^ = 0%, Fig. 2).

### Clinical outcomes

**Fig. 3:**
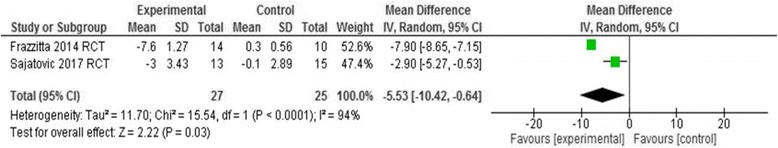
Summary effect sizes for outcome of change in MDS-UPDRS motor score. Green squares indicate individual SES. Black colored diamond indicates the summary effect size; *RCT* randomized clinical trial, *SD* standard deviation, *CI* Confidence Interval, *I*^2^ statistic to determine heterogeneity, *Z* z-score

MDS-UPDRS-III motor examination scores were reported in 2 RCTs [56, 57] (*N* = 52) and pooling resulted in a significant heterogeneous SES (MD -5.53, 95% CI -10.42 to -0.64; Z = 2.22, *P* = 0.03, I^2^ = 94%, Fig. 3). All studies noted statistically significant improvements for clinical outcome measures [56, 57, 60–63] (Table 1).

Few studies conducted statistical analysis between exercise-induced BDNF blood concentration and scores on clinical outcome measures. Marusiak et al. [62] found a statistically significant association between interval training induced increases in BDNF concentration and decrease in Parkinsonian rigidity. The study by Fontanesi et al. [60] found a statistically significant association between inpatient rehabilitation induced increases in TrkB signaling in the lymphocytes and improvement in MDS-UPDRS total and MDS-UPDRS-II score. The trial by Frazzitta et al. [56] found no correlation between BDNF blood levels and the MDS-UPDRS motor examination score (Part III).

### Methodological quality

A kappa statistic, which accounts for chance agreements between the two raters, was .62 for the trials by Frazzitta et al. [56] and Sajatovic et al. [57], .55 for the studies by Fontanesi et al. [60], Angelucci et al. [63] and Zoladz et al. [61], and .57 for the study by Marusiak et al. [62]. The percent agreement between the two raters was 72% to 77% (Additional file 1).

Strengths towards the internal validity of the studies included use of masked assessors, and administration of valid and reliable outcome measures. Four studies [56, 57, 60, 61] used assessors masked during the clinical outcomes testing, including the two randomized controlled trials [56, 57]. Five studies administered outcome measures that were valid, reliable and assessed consistently across all study participants [56, 57, 60–62]. Three studies reported loss to follow-up after baseline testing, which was less than 20% [60–62].

Deficiencies included the lack of an a-priori justification for the sample size needed to detect an exercise-induced effect on BDNF levels [60–63], and lack of masking of the assessor conducting the BDNF assays [57, 60–63]. Additional deficiencies noted were lack of description of adherence to the intervention, short duration of training and short follow-up, lack of details about method used for participant recruitment into study protocol, failure to characterize the cognitive status of patients, failure to describe adverse events, and failure to recruit younger age patients.

Each ‘deficiency’ noted above represents an opportunity for future research and discovery. For example, age and physical exercise intensity may be a rate limiting factor in activity-dependent BDNF neuroplasticity. To address patient age, a future study on the effect of physical exercise on BDNF concentration could compare the response to exercise by age group because younger patients with PD typically display greater baseline physiologic reserve (e.g., VO_2_ Maximum) than older patients with PD, and may be able to sustain physical exercise at higher physiologic intensities than older patients.

### Studies BDNF Assays Procedures

BDNF assay procedures were reported by all studies. Sajatovic et al. [57] did not report if the blood sample collection was obtained in the morning or later during the day. Plasma samples of BDNF were assayed by using ELISA per manufacturer instructions (Quantikine® ELISA Human BDNF Immunoassay; R&D Systems, Minneapolis, MN, USA). Frazzitta et al. [56] did not report blood sample collection time but reported that serum BDNF concentrations were evaluated in a capture ELISA according to the protocol provided by the manufacturer without including the kit manufacturer information. Marusiak et al. [62] and Zoladz et al. [61] assayed serum BDNF from morning blood samples with an ELISA Kit (Promega, Wallisellen, Switzerland) after appropriate dilution with Block and Sample solution (provided with the kit). Angelucci et al. [63] analyzed serum BDNF from blood samples that were obtained in the morning. Sandwich ELISA (R&D Systems, USA; cat. No. DY248) was used for BDNF assays according to the manufacturer’s instructions. Fontanesi [60] used morning blood samples for the BDNF assays by EDTA Western Blotting.

## Discussion

The present systematic review and meta-analysis is the first to show aggregated evidence that physical exercise training increases BDNF blood levels in human PD. The main finding is that, in line with most pre-experimental studies that report significant results, pooling of the two available RCTs showed a significant SES in favour of physical exercise training for increasing BDNF levels. The BDNF results are paralleled by concomitant reductions in motor symptoms (UPDRS-scores), confirming possible effects on the dopaminergic pathways. Although our synthesis results support that controlled physical exercise training can have a positive impact on BDNF levels, and the number of publications in human PD is increasing, this review remains limited to a small number of studies that reported BDNF with few participants. Nevertheless, this is, to our knowledge, the first research synthesis study to evaluate the effects of controlled physical training trials on BDNF levels in human PD.

Our BDNF results are in line with prior human research in a variety of psychiatric conditions, and a recent review of neurotrophic factors in animal models of exercise and Parkinson’s disease [42]. Studies utilizing psychiatric conditions have reported clinical improvements with increased serum BDNF levels following non-pharmacological approaches, including exercise [64, 65], computer-assisted cognitive enhancement in schizophrenia [35] and mindfulness clinical trials in bipolar-disorder [66]. However, caution is warranted when extrapolating the results from psychiatric conditions to Parkinson’s disease because clinical measures used in psychiatric conditions are different from the UPDRS.

Collectively, our meta-analysis found a SES of -5.53 point improvement on the motor examination part of the UPDRS, which is beyond the minimal clinically important difference [67]. Data from the included studies on exercise-induced increases in BDNF blood concentration rarely correlated with clinical outcome measures [60, 62]. Studies are needed to determine the clinical relevance of exercise-induced increases in BDNF blood levels.

The most appropriate mode and intensity of exercise to achieve gains in BDNF concentrations in human PD remains controversial. For example, the 4-week intensive rehab training in the RCT of Frazzitta et al. [56] contained one hour of balance exercises and treadmill cue training, embedded in a daily three-hour general rehabilitation program but there is no information on duration of each sub-part. In contrast, Sajatovic et al. [57] administered fast-paced, low-resistance cycling for 20 minutes followed by resistance training for 20 minutes using a progressive sequence of resistance band, 3 times a week for 12 weeks [57]. These are quite different modes and intensity of exercise training but apparently give similar, favorable results on blood BDNF concentration.

The kappa value of .55 to .57, for the four uncontrolled studies and the kappa value of .62 for the two RCTs indicates fair to good agreement. The kappa value suggests that the raters accounted for 55% to 62% of the agreement over and above what would be expected by chance alone [55]. The percent exact agreement between the two raters -- those instances for which both raters agreed that a study fulfills a methodological quality criteria – was 72% to 77%, indicating raters agreed on over two-thirds of methodological quality items.

The evidence presented here is preliminary and does not address several important issues inherent to BDNF blood levels testing. BDNF has attracted increasing interest as potential biomarker to support the diagnosis or monitor the efficacy of therapies in brain disorders [64, 68]. Circulating BDNF levels can be measured in serum, plasma or whole blood. However, the use of BDNF as biomarker is limited by the probable poor reproducibility of results, likely due to the variety of methods used for sample collection and BDNF analysis, as well as the possible variations among performance for the different ELISA kits in term of intra-assay variation, inter-assay variation, detection range, and sensitivity [69]. In addition to the technical and methodological issues discussed here, several studies report that the socio-demographic determinants and other factors may affect serum levels of BDNF such as gender [70], age [71], body mass index [72], and disease status [69, 73]. Several studies have suggested an interaction, with respect to circulating BDNF, between gender and age [70, 71, 74]. Meta-analyses and reviews of clinical studies based on the measurement of BDNF in whole blood, serum, or plasma have reported significantly lower BDNF levels at diagnosis in patients with mental illnesses [73–75]. These reviews however, highlighted severe discrepancies among studies, which even reported opposed results (increase versus decrease, or no change).

BDNF levels have also demonstrated to be affected by pharmacological treatments including antidepressant treatments [64, 75]. Regarding description of pharmacologic treatments in the included studies, only the study by Frazzitta et al. [56] stated that, in order to minimize a possible polypharmacy effect, participants were enrolled if they were currently taking rasagiline monotherapy. In the studies by Fontanesi et al. [60] and Frazzitta et al. [56], patients remained on their pharmacotherapy regimen throughout the study duration. Sajatovic et al. [57] reported that patients were on a stable dose of levodopa medication throughout the trial. Regarding enrolment of participants on anti-depressant medication, the trial by Sajatovic et al. [57] included participants who were on a stable dose of anti-depression medication for at least 1 month prior to trial enrolment. The trial by Frazzitta et al. [56] excluded patients who were on anti-depressant medication. The studies by Fontanesi et al. [60] Zoladz et al. [61] and Marusiak et al. [62] did not explicitly provide details about participants’ anti-depression medication.

BDNF variability of response may relate, in part, to age, sex, medication and dietary factors, disease duration, cognitive status, air quality, or genetic factors [60, 76–82]. Studies have also shown differences in BDNF laboratory sample collection kits that may add an additional variability [83]. Concrete improvements to address variability in BDNF response may include adoption of repeated measures designs in which the sample is collected repeatedly from the same subject over time, pre and post training intervention (which would allow for within-subject comparisons), or use of surrogate markers of BDNF action (such as tyrosine receptor kinase signalling) as demonstrated in the forward thinking study by Fontanesi [60]. Additional improvements to decrease BDNF variability may include sampling from jugular vein catheters (instead of peripheral veins), or from saliva [82], directly after an exercise session.

### Limitations

The main limitation of this review is the small number of papers that were available for inclusion. Although our results are based on a small number of studies, the participant characteristics were relatively homogenous in terms of years of age, Parkinson’s disease stage, years since diagnosis, and ambulatory status. The studies were also homogenous in the adoption of cycling training mode, administration of the MDS-UPDRS and the pharmacologic characteristics of the participants [57, 60–63]. Our study is limited to the focus on one neurotrophic factor, exercise-induced changes in BDNF concentrations in human PD, and not other neurotrophic factors, which limits the generalizability to BDNF only. Although there are a number of potential opportunities for including BDNF as a clinical marker of brain health in PD [81], including outcome prediction [82], and/or development of physical exercise treatment interventions [84], further studies and methodological evaluations need to take place to standardize BDNF measurement and evaluate its usefulness as a clinical marker of brain health in PD.

The Cochrane Library Guidelines do not recommend meta-analysis when the designs of the studies are too different, if the outcomes measured are not sufficiently similar, or if there are concerns about the quality of the studies, for an average result across the studies to be meaningful (for review, see http://www.cochranelibrary.com/about/about-cochrane-systematic-reviews.html). Meta-analysis was a small part of the current review. We included all human studies, including non-randomized controlled studies (except case reports/single case studies as per exclusion criteria), covering the entire body of literature.

We emphasize that the small number of available studies is a limitation and further research is urgently needed to provide a realistic evaluation of the possible effects of exercise training on BDNF of PD patients. Prior international systematic reviews have evaluated effects of exercise training on BDNF and cognition (e.g., Alzheimer’s) and usually these synthesis reports also have had a small number of studies (between 6-8) [85].

We propose that strengths of the current preliminary meta-analysis and systematic review include: a) the manuscript addresses a clinically important, understudied area of neurorestorative rehabilitation research, and b) the results challenge and seek to shift current research and clinical practice paradigms by extending novel theoretical concepts of physiologic use of exercise on neuroplasticity in ageing human brain to humans living with Parkinson’s disease.

## Conclusions

In summary, the presented results provide preliminary evidence of an exercise-induced increase in BDNF blood levels in human PD. Further high-quality, rigorously conducted randomized clinical trials of physical exercise effect on BDNF blood levels are needed to show robustness of the presented optimistic trend and to determine the neuroplastic mechanisms (for review, see [86]) that link BDNF blood levels, physical exercise, and functional outcomes in PD.

## Additional file

Additional file 1: Fulfilled items of methodological quality plus quality criteria for randomized controlled trials (RCT) and noncontrolled studies. All studies were scored on items concerning ‘internal validity’, ‘descriptive criteria’ and ‘statistical criteria’. The NHLBI Appraisal tool to evaluate RCTs consists of nine criteria for internal validity, two for descriptive criteria and three for statistical criteria. The NHLBI Appraisal tool to evaluate uncontrolled pre-post studies consists of four criteria for internal validity, five criteria for descriptive criteria, and three for statistical criteria. The tool to evaluate case control studies consists of two criteria for internal validity, six descriptive criteria and one statistical criteria. (DOCX 15 kb)

## Abbreviations

PD: Parkinson’s disease
BDNF: brain-derived neurotrophic factor
TH: tyrosine hydroxylase
MPP+: 1-methyl-4-phenylpyridinium
CREB: cyclic AMP response element-binding protein
MDS: Movement Disorder Society
PRISMA: Preferred Reporting Items for Systematic Reviews and Meta-Analyses
MD: mean difference
SMD: standardized mean difference
SDi: population standard deviation
SES: summary effect size
BDNF-TrkB: brain-derived neurotrophic factor tyrosine receptor kinase signaling
ELISA: enzyme-linked immunosorbent assay
