# Supplementary material for: Exercise-induced increase in brain-derived neurotrophic factor in human Parkinson's disease: a systematic review and meta-analysis
Source: Transl Neurodegener. 2018 Mar 20;7:7. doi: 10.1186/s40035-018-0112-1 (PMC5859548; doi:10.1186/s40035-018-0112-1)
Supplement: Supplementary file 1 — Fulfilled items of methodological quality plus quality criteria for randomized controlled trials (RCT) and noncontrolled studies. All studies were scored on items concerning ‘internal validity’, ‘descriptive criteria’ and ‘statistical criteria’. The NHLBI Appraisal tool to evaluate RCTs consists of nine criteria for internal validity, two for descriptive criteria and three for statistical criteria. The NHLBI Appraisal tool to evaluate uncontrolled pre-post studies consists of four criteria for internal validity, five criteria for descriptive criteria, and three for statistical criteria. The tool to evaluate case control studies consists of two criteria for internal validity, six descriptive criteria and one statistical criteria. (DOCX 15 kb) [file 40035_2018_112_MOESM1_ESM.docx]

| Appendix – Fulfilled items of methodological quality as proposed by Lim [54]. | | | | | | | |
| --- | --- | --- | --- | --- | --- | --- | --- |
| First author | Design | Items positively scored on criteria for “internal validity”  e.g., b_1_,b_2_,f,g,h_1_,h_2_, j_1_, j_2_, j_3_, k_1_, k_2_, k_3_, k_4_, | Items positively scored on “descriptive criteria”  e.g., a_1_, a_2_, a_3_, a_4_, a_5_, c, d, e_1_, e_2_, e_3_, e_4_, i, | Items positively scored on “statistical criteria”  e.g., l_1_, l_2_, m, n_1_, n_2_, n_3_, n_4_ | Quality rating | Kappa rating [55] | Rater one versus two  Exact agreement  (%) |
| Sajatovic [57] | RCT | h_2_, j_2,_ k_1_ | a_2_, c | l_1_, n_1_ | poor | .622 | 73.0% |
| Frazzitta [56] | RCT | b_1_, b_2_, g, h_2_, j_2_, k_1_, k_2_ | a_2_, c | n_1_ | fair |  |  |
| Marusiak [62] | Case series | k_4_ | a_3_, a_4_, a_5_, e_4_, i | n_4_ | fair | .571 | 77.7% |
| Angelucci [63] | Pre-post | j_1_, j_2_, k_3_ | a_1_, a_3_, a_5_ | n_2_ | fair | .554 | 72.2% |
| Fontanesi [60] | Pre-post | h_2_, j_2_, k_3_ | a_1_, a_3_, | n_2_ | fair |  |  |
| Zoladz [61] | Pre-post | h_2_, j_1_, j_2_, k_3_ | a_3_, a_5_, d | n_2_ | fair |  |  |
| Table note: RCT, randomized controlled trial; Pre-post pretest posttest; All studies were scored on items concerning ‘internal validity’, ‘descriptive criteria’ and ‘statistical criteria’. The NHLBI Appraisal tool to evaluate RCTs consists of nine criteria for internal validity, two for descriptive criteria and three for statistical criteria. The NHLBI Appraisal tool to evaluate uncontrolled pre-post studies consists of four criteria for internal validity, five criteria for descriptive criteria, and three for statistical criteria. The tool to evaluate case control studies consists of two criteria for internal validity, six descriptive criteria and one statistical criteria.  An ‘a1’ indicates a positive score on a description of the eligibility criteria, ‘a2’ means the study was described as a randomized controlled trial, ‘a3’ means that the study question or objective were clearly stated, ‘a4’ means the intervention procedures were clearly defined, ‘a5’ means intervention procedures were clearly described and the procedures were delivered consistently across the study population; ‘a6’ means the study population was clearly described, including a case description; ‘b1‘ means that the method of randomization was adequate, whereas ‘b2,‘ means that the treatment allocation was concealed; ‘c’ indicates that groups were similar at baseline; ‘d’ means that the sample in the study was representative of the clinical population of interest; ‘e1’ indicates that all participants that met inclusion criteria were enrolled, ‘e2’ subjects were enrolled consecutively, ‘e3’ means enrolled subjects were comparable; ‘e4’ means cases were enrolled consecutively; ‘f ’ indicates that other interventions were avoided or similar in the groups; ‘g’ means that there was high adherence to the intervention for each treatment group; ‘h1’ means that the study participants and providers were masked for allocation, whereas ‘h2’ means that the assessors were masked to the participants’ group assignment; ‘i’ means the results were well described; ‘j1’ means that the outcome measures were prespecified and clearly defined, ‘j2’ means that the outcome measures were valid, reliable, and assessed consistently across all study participants, whereas ‘j3’ means that the outcome measures of interest were taken multiple times before the intervention and multiple times after the intervention; ‘k1’ means that the overall dropout rate from the study at endpoint was 20% or lower of the number allocated to treatment, ‘k2’ means that the differential drop-out rate (between treatment groups) at endpoint was 15 percentage points or lower, ‘k3’ means that the loss to follow-up after baseline was 20% or less and those lost to follow-up where accounted for in the statistical analysis, whereas ‘k4’ means the length of follow-up was adequate; ‘l1’ means that the sample size was sufficiently large to be able to detect a difference in the main outcome between groups with at least 80% power, whereas ‘l2’ means the sample size was sufficiently large to provide confidence in the findings; ‘m’ means that an intention-to-treat analysis was applied; ‘n1’ means that outcomes reported or subgroups analyzed were prespecified (i.e., identified before analyses were conducted), ‘n2’ means that the statistical methods examined changes in outcome measures from before or after the intervention and statistical tests were done that provided p values for the pre-to-post changes, ‘n3’ means that if the intervention was conducted at a group level, the statistical analysis took into account the use of individual-level data to determine effects at the group level, ‘n4’ means the statistical methods were well described. Criteria ‘a2’, ‘b1’, ‘b2’, ‘c’, ‘f’ ‘g’, ‘h1’, ‘h2’, ‘J2’, ‘k1’, ‘k2’, ‘m’, and ‘n1’ were scored for controlled trials [56, 57]. Criteria ‘a1’, ‘a3’, ‘a5’, ‘d’, ‘e1’, ‘e2’, ‘h2’ ‘j2’, ‘j3’, ‘k3’, ‘l2’, ‘n2’, and ‘n3’ were scored for pre-experimental studies with an uncontrolled pre-post design [60, 61, 63]. Criteria for ‘a3’, ‘a4’, ‘a5’, ‘a6’, ‘e3’, ‘e4’, ‘i’, ‘k4’, and ‘n4’ were used for the case series design [62]. | | | | | | | |
